# Landscape and evolutionary trends of nanotechnology in cancer radiosensitization

**DOI:** 10.1186/s11671-026-04836-8

**Published:** 2026-08-04

**Authors:** Miao Yan, Yuanchao Cheng, Quan Li, Xvdong Zhou, Chao Ren, Guilong Zhang, Xicheng Song

**Affiliations:** 1 Department of Otorhinolaryngology, Head and Neck Surgery, Yantai Yuhuangding Hospital, Qingdao University, No.20, East Road, Zhifu District, Yantai, 264000 China; 2 Shandong Provincial Key Laboratory of Neuroimmune Interaction and Regulation, Yantai Yuhuangding Hospital, Yantai, China; 3 Shandong Provincial Clinical Research Center for Otorhinolaryngologic Diseases, Yantai Yuhuangding Hospital, Yantai, China; 4 Yantai Key Laboratory of Otorhinolaryngologic Diseases, Yantai Yuhuangding Hospital, Qingdao University, Yantai, China; 5 Department of Neurology, Yantai Yuhuangding Hospital, Qingdao University, Yantai, China; 6 School of Pharmacy, Shandong Technology Innovation Center of Molecular Targeting and Intelligent Diagnosis and Treatment, Shandong Medical and Pharmaceutical University, No. 346, Guanhai Road, Laishan District, Yantai, 264003 China

**Keywords:** Nanoradiosensitizers, Radiotherapy, Immunotherapy, Tumor microenvironment, Immunogenic cell death

## Abstract

Radiotherapy is one of the most basic clinical tumor treatment methods, yet its efficacy is often limited by radioresistance and off-target toxicity. Nanoradiosensitizers have emerged as a promising strategy for enhancing local dose deposition and regulating tumor microenvironment (TME). Despite the rapid development of the nanoradiosensitizer field, a comprehensive roadmap describing its evolution remains lacking. We analyzed 1,866 publications on nanoradiosensitizers in the Web of Science Core Collection (WoSCC) database up to 2024. The global landscape is drawn using advanced visualization tools, including Bibliometrix, VOSviewer, CiteSpace, and Scimago Graphica. Our analysis reveals that the field has undergone exponential growth and a distinct paradigm shift, with research focus evolving from initial material synthesis (e.g., high-Z metals for physical dose enhancement) to complex biological regulation (e.g., hypoxia relief, TME regulation). Notably, keywords and trend analysis have identified that the integration of nanoradiosensitizers with immunotherapy has become the frontier of the current research field, such as enhanced immunogenic cell death (ICD) and immune checkpoint blockade (ICB). Overall, this study highlights the shift of nanoradiosensitizers from material-driven exploration toward mechanism-based biological and translational studies. Future research must prioritize overcoming translational barriers, including biosafety, large-scale manufacturing, and clinical evaluation, to enable the clinical application of radioimmunotherapy strategies.

**Graphical abstract:**

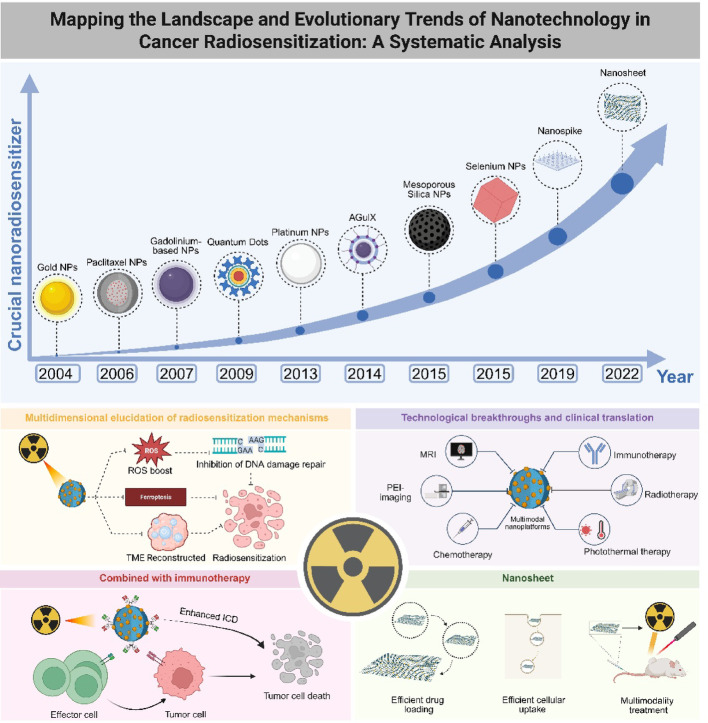

**Supplementary Information:**

The online version contains supplementary material available at 10.1186/s11671-026-04836-8.

## Introduction

Malignant tumors remain a formidable challenge to global health. According to the latest statistics from the American Cancer Society, there were approximately 19.9 million new cancer cases and 9.7 million cancer-related deaths worldwide in 2024 [1, 2]. Radiotherapy is a mainstay of oncology treatment, with approximately 50% of cancer patients receiving radiotherapy [3]. Radiotherapy relies on high-energy ionizing radiation to trigger reactive oxygen species (ROS) that directly or indirectly induce DNA damage [4]. Although radiotherapy offers unique advantages such as precise target location, controllable dose delivery and deep tissue penetration [5], its clinical efficacy is often compromised by intrinsic radioresistance driven by the hypoxic tumor microenvironment (TME) [6–8].

In recent years, the intersection of nanotechnology and radiation oncology has opened new avenues for the development of nanoradiosensitizers [9–11]. In particular, nanomaterials containing high atomic number (high-Z) elements (e.g., gold (Au) [12], platinum (Pt) [13], hafnium oxide HfO₂ [14], tungsten oxide WO₃₋ₓ [15, 16], nanoscale metal–organic frameworks (nMOFs) [17–20]) can be used as radiosensitizers to amplify radiation energy deposition within tumors. A landmark milestone in this field is NBTXR3 (Hensify®), a HfO₂-based radiosensitizer that obtained CE marking in 2019 for the treatment of soft-tissue sarcoma, validating the clinical feasibility of nanoradiosensitizers [21]. Beyond physical dose enhancement, recent innovations have introduced a photosensitizer-conjugated nanoscintillator systems, which enable X-ray-induced photodynamic therapy (X-PDT) in deep tissues, representing a paradigm shift in multimodal combination therapy based on traditional physical radiosensitization [22–27].

With the rapid development and advancement of nanoradiosensitizer research, it is particularly important to systematically explore the evolutionary trajectory of this field by identifying research hotspots and capturing emerging frontiers. Bibliometric analysis provides a powerful quantitative approach for drawing knowledge maps in specific fields [28, 29]. Although bibliometrics has been applied in the field of nanomedicine [30–34], a comprehensive visualization of the global research landscape on nanoradiosensitizers is still lacking.

Therefore, this study conducted a systematic quantitative analysis of scientific research output in the field of nanoradiosensitizers from 2006 to 2024. We not only focused on the overall evolutionary trajectory of global development trends, but also identified high-impact countries/regions, institutions, and research scholars. Through cluster analysis and burst detection, this study revealed the evolution of nanoradiosensitizers from material-driven exploration to biological mechanism-based transformation research, and predicted emerging frontiers and potential research hotspots. This study provided a valuable reference for researchers to find potential breakthroughs in the field of cancer radiosensitizers.

## Methods

### Data source and search strategy

The English-language literature published between January 1, 2006 and December 31, 2024 was retrieved from the Web of Science Core Collection (WoSCC) database. All bibliographic records and citation data were downloaded from the WoSCC on August 21, 2025. The literature search ends on December 31, 2024, to ensure a complete and stable dataset for the full calendar year, avoiding the volatility of more recent, incomplete indexing. WoSCC was selected as the primary data source due to its inclusion of high-impact, peer-reviewed journals widely recognized in bibliometric research [32, 35–37].

To ensure maximal coverage of literature related to nanomaterials in radiotherapy sensitization, the following combination of topic (TS) terms was employed: (TS = (“radiosensitizer” OR “radiosensitizers” OR “radiosensitization” OR “radiosensitising” OR “radiosensitizing” OR “radio-sensitizer” OR “radio-sensitizers” OR “radio-sensitization” OR “radio-sensitising” OR “radio-sensitizing” OR “radiation sensitizer” OR “radiation sensitizers” OR “radiotherapy sensitizer” OR “radiotherapy sensitizers” OR “radiation dose enhancement” OR “radiotherapy enhancement” OR “nanoparticle radiosensitizers” OR “X-ray sensitizer” OR “X-ray sensitizers”)) AND (TS = (nano*)) NOT TS = (nanomolar).

**Fig. 1:**
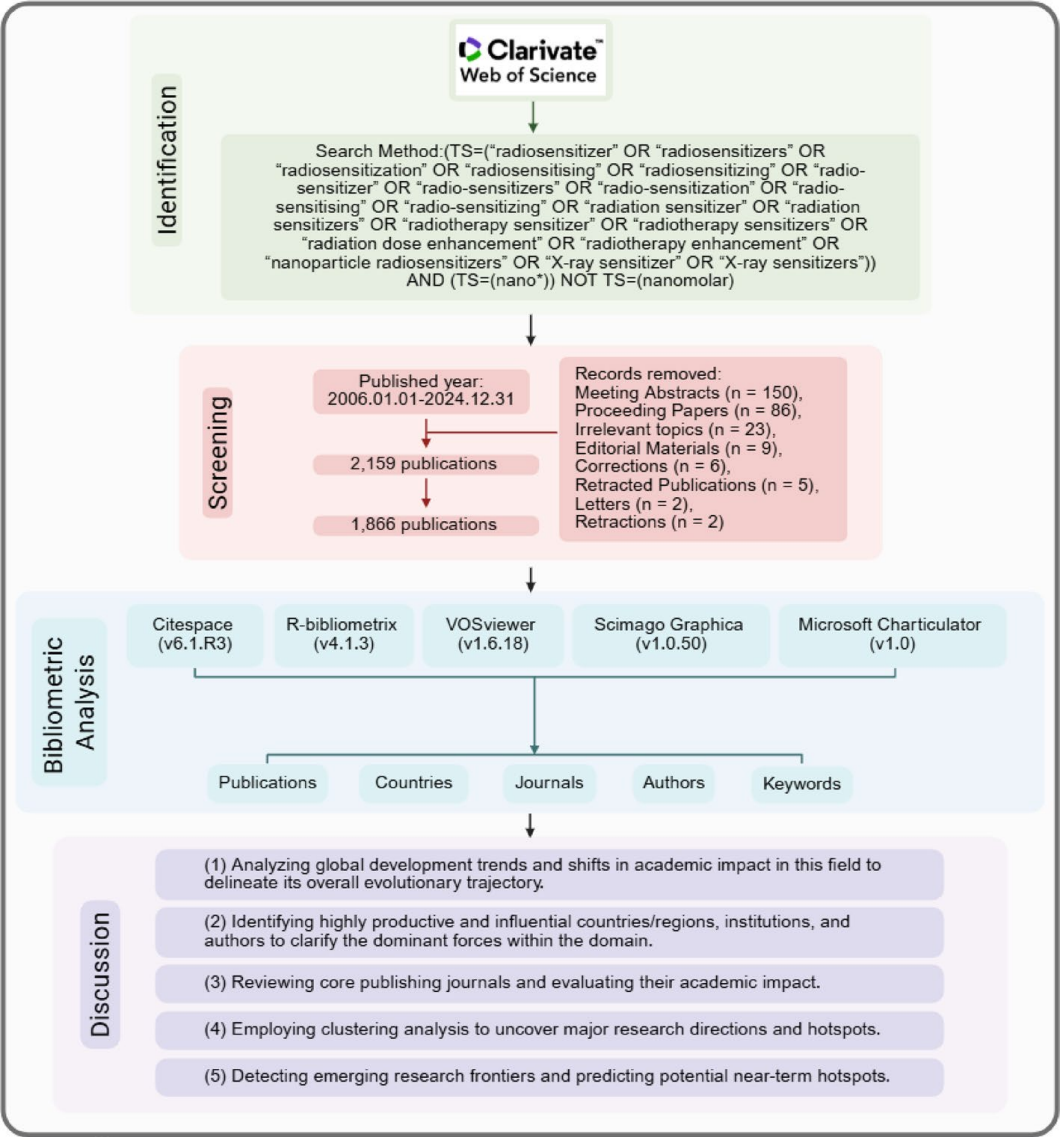
Flowchart of the literature screening process

All eligible records “Full Record and Cited References” option was explicitly selected to ensure the acquisition of complete bibliographic metadata and citation network information. The complete literature search and processing workflow of this study has been clearly illustrated in Fig. 1.

### Data analysis and visualization

To systematically present a comprehensive landscape of researches on nanomaterials in radiotherapy sensitization, this study employed an integrated suite of bibliometric tools, including R (v4.1.2) with bibliometrix package (v3.0.3), CiteSpace (v6.1.R2), VOSviewer (v1.6.18), Scimago Graphica (v1.0), and Microsoft Charticulator (v1.0) [35–37]. Through the synergistic application of these platforms, we made quantitative characterization of the fundamental features of the included literature, including annual outputs, country/regional contributions, core research institutions, active authors, and source journal distributions. More importantly, through in-depth analyses such as keyword co-occurrence and clustering, we systematically identified research hotspots, knowledge structures, and dynamic evolutionary trends in the field. This multi-tool complementary analytical strategy is designed to comprehensively and multidimensionally reveal the developmental trajectory of the discipline across macro, meso, and micro levels.

During the R-bibliometrix analysis, we quantified the publication output and citation counts of authors, journals, institutions, and countries, presenting the top 20 results for each metric. Country/region productivity was assessed using the AU1_CO field generated by bibliometrix/Biblioshiny, which assigns each publication to the country or region of the first author and counts it once. Institutional and author productivity analyses were conducted using full counting. International collaboration analyses were based on all-author country affiliations and co-authorship links and were used to characterize collaborative relationships rather than single-country productivity. Simultaneously, a word cloud of author keywords was generated using the package, with the top 50 high-frequency terms selected—their font sizes being proportional to their occurrence frequency. The analysis utilized Biblioshiny, the interactive visualization interface of bibliometrix, to further display data results and their social network structure.

Institutional names were manually checked and standardized to reduce inconsistencies caused by abbreviations, spelling variants, capitalization, transliteration, and country-specific suffixes. Obvious variants referring to the same institution were unified under a standardized institutional name. Organization-level entities indexed by WoSCC (e.g., Chinese Academy of Sciences and University of Texas System) were retained as recorded. For author analyses, highly productive authors were manually cross-checked using affiliation information and research topics where necessary. Ambiguous author names were not merged unless sufficient evidence supported their identity.

VOSviewer was employed to construct and visualize collaborative networks. We generated network maps for co-authorship (countries/regions, and institutions) and keyword co-occurrence. In these visualizations, node sizes represent the frequency of occurrence or citation counts, while the thickness of connecting lines reflects the strength of collaborative relationships.

We utilized CiteSpace to conduct institutional collaboration analysis, subject category co-occurrence analysis, and reference co-citation analysis, as well as to identify references or keywords exhibiting “burst” influences within specific time periods. Furthermore, the software's unique dual-map overlay analysis module visualizes knowledge flow, citation pathways, and the thematic distribution of academic journals [38]. The specific parameters were set as follows: time slicing = 2 years; selection criteria = g-index (k = 25); link retention factor (LRF) = 3; look back years (LBY) = 5, network pruning = minimum spanning tree and pruning sliced networks.

Scimago Graphica was utilized to construct scientific research collaboration networks among countries. Additionally, Microsoft Charticulator was employed to create chord diagrams, providing an intuitive visualization collaborative relationships among different countries/regions.

## Results

### Analysis of annual publication and citation trends

A total of 2,159 publications on nanoradiosensitizers were retrieved from the WoSCC (2006–2024) (Fig. 1). After rigorous screening to exclude non-article publications (e.g., meeting abstracts, proceedings) and irrelevant topics, 1,866 publications were included for final analysis (Fig. 1). This corpus comprised of 1,631 original articles (87.4%) and 235 review articles (12.6%). The total citation count for these publications was 61,721, with an average of 33.08 citations per item.

**Fig. 2:**
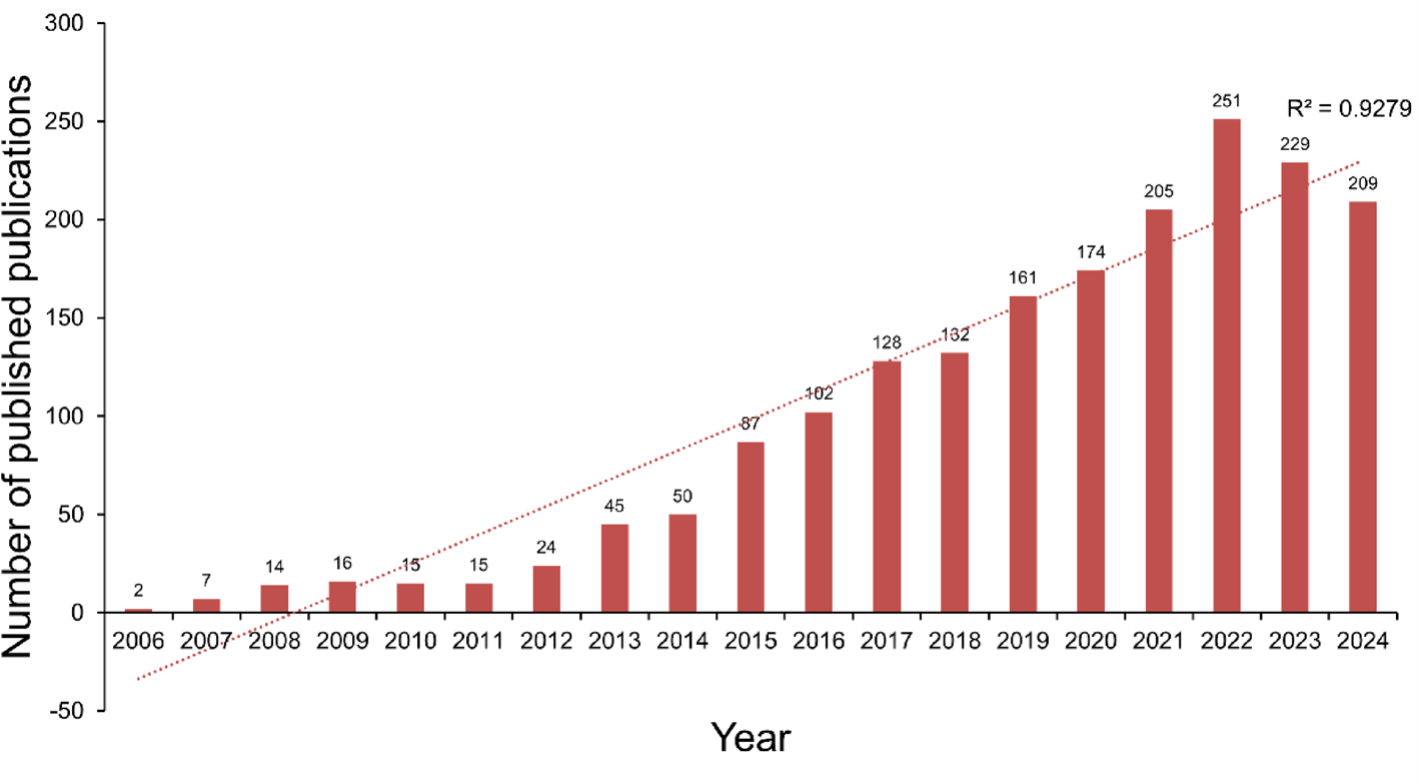
The global annual number of publications

The annual publication output from 2006 to 2024 is presented in Fig. 2. The field of nanoradiosensitizers has witnessed substantial growth, with the cumulative total of publications rising from 2 in 2006 to 1,866 by the end of 2024, at an average annual growth rate of 29.47% (Fig. 2). The year 2022 was the peak of annual publications, with 251 articles. Curve fitting analysis confirmed a statistically significant upward trend (R = 0.9279) (Fig. 2). As shown in Figure S1, publications published between 2012 and 2023 accumulated more than 3,000 citations each by the time of data retrieval. Publications published in 2017 received the highest total citation count (9,487 citations). The lower citation count observed in 2024 may attributable to their shorter citation accumulation period. These metrics collectively indicate that nanoradiosensitizers have evolved into a highly active research domain within oncology.

### Contribution of countries/regions

**Fig. 3:**
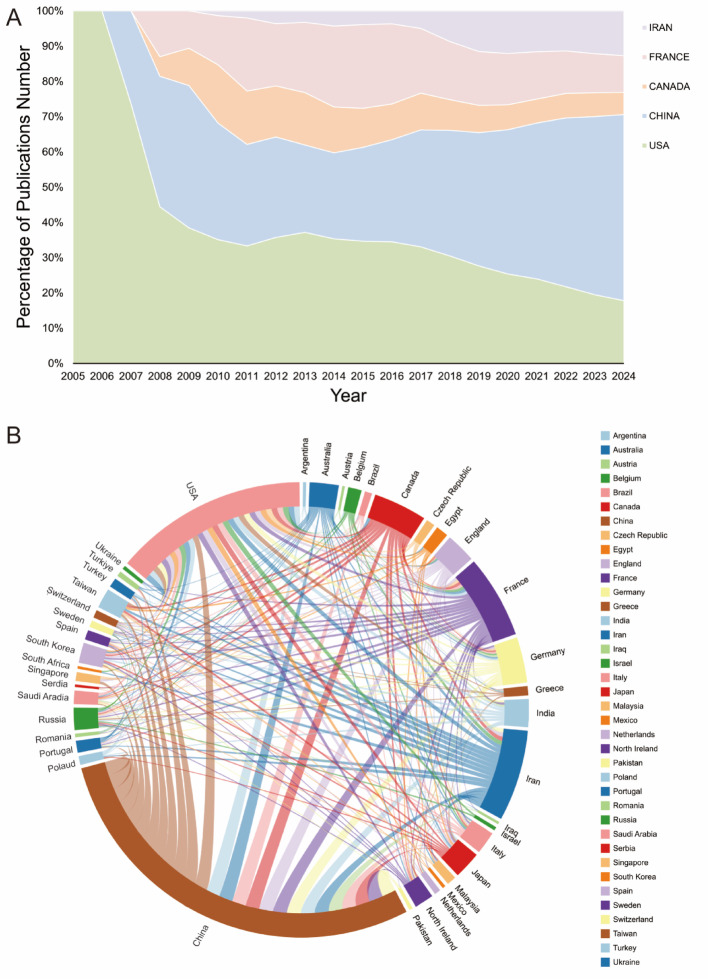
**A** Relative proportions of annual publications for the top 5 countries; **B** Collaboration heatmap. Line thickness reflects the strength of international cooperative links

The publications originated from 64 countries/regions. China emerged as the leading contributor (774 publications, 41.5%), followed by the USA (236, 12.6%), Iran (161, 8.6%), France (115, 6.2%), and Canada (85, 4.6%). Figure 3A illustrates the trend of publication share over time. The results showed that the United States developed the earliest in this field; since 2008, the share of Chinese publications gradually increased and surpassed that of the United States in 2018 (Fig. 3A). Since then, it maintained a rapid growth trend until 2024 (Fig. 3A).

**Fig. 4:**
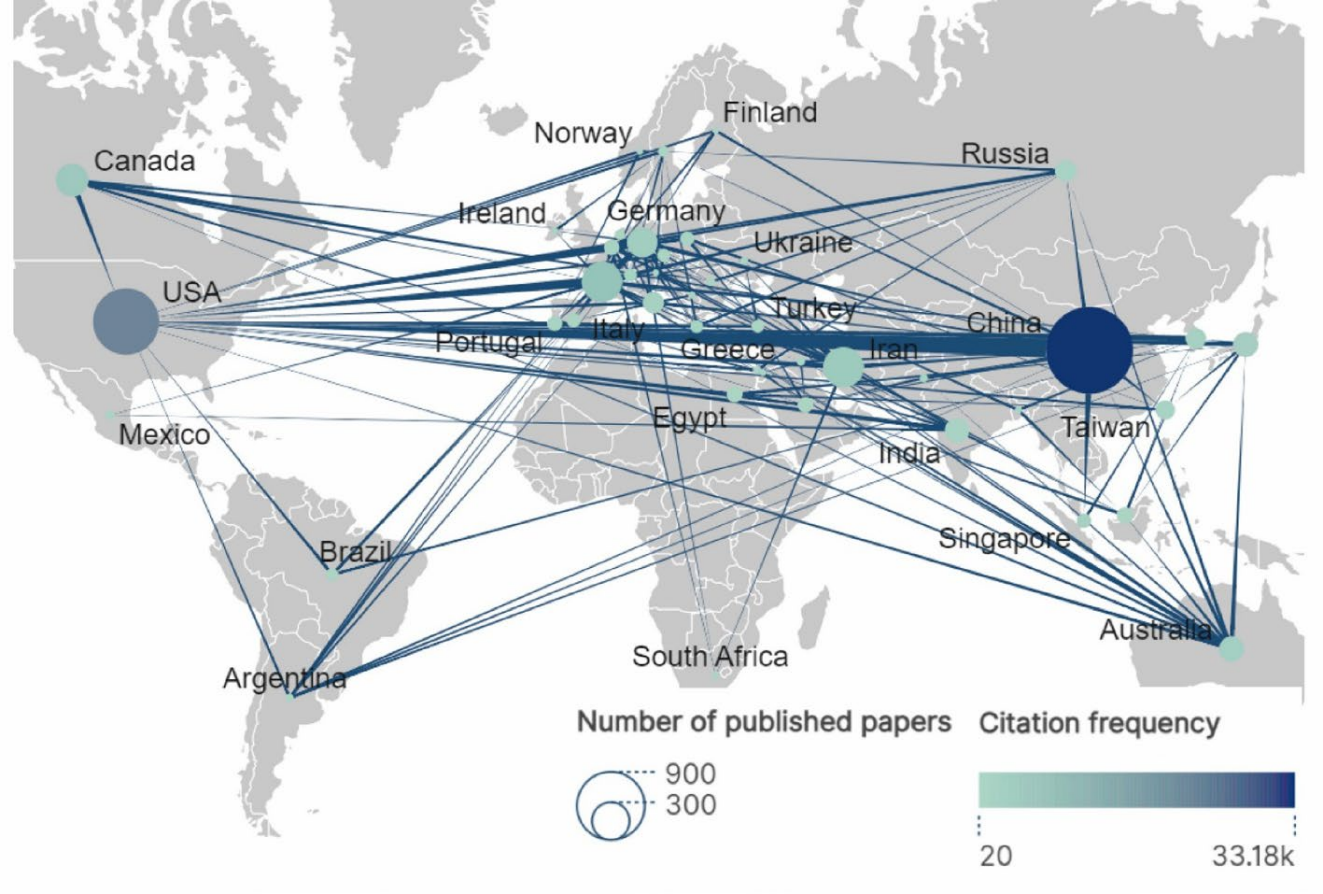
Geothermal map of international cooperation. Node size and color correspond to the publication volume; line thickness reflects collaborative strength

International collaboration analysis (Figs. 3B and 4) revealed that China maintains its strongest partnerships with the United States, Singapore, and Portugal. Network visualization (Figure S2, threshold ≥ 5 publications) delineated five distinct clusters. The largest cluster (red) centered on China showed the most extensive collaborative links, followed by the cluster (green) centered on the United States (Figure S2). This network topology confirms that China and the United States serve as the primary hubs driving global research in this field.

### Contribution of institutions

**Fig. 5:**
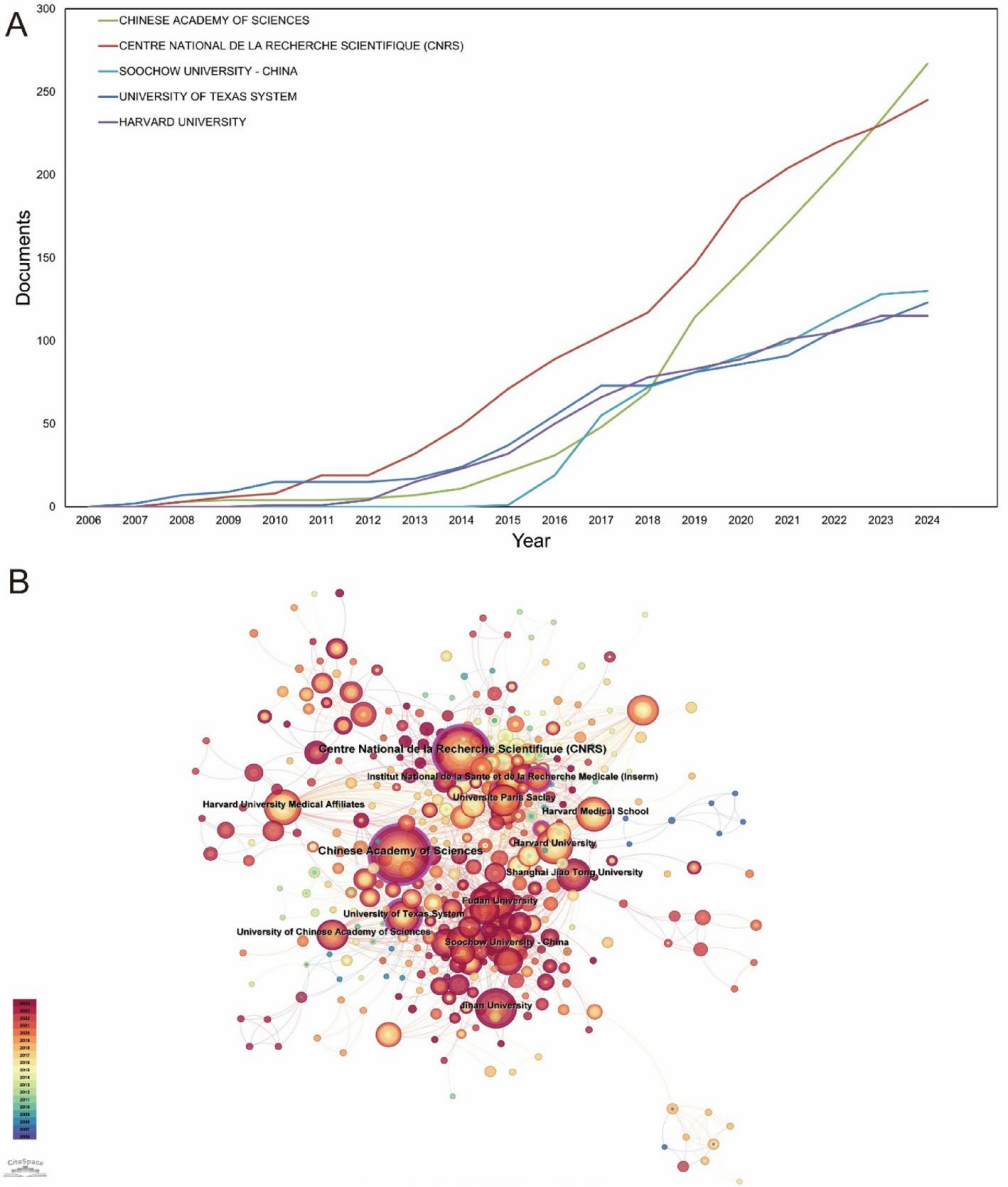
**A** Trends in relative publication proportions for the top 5 institutions. **B** Institutional collaboration analysis by CiteSpace

Figure 5A shows the top five high-output institutions: the Chinese Academy of Sciences (CAS), the Centre National de la Recherche Scientifique (CNRS), Soochow University, the University of Texas System, and Harvard University. Among them, CNRS has maintained relatively stable output, while the annual output of CAS has exceeded that of CNRS since 2023, reflecting the active trend of China’s scientific research activities in this field (Fig. 5A). The institutional collaboration network (Fig. 5B) further confirms the central position of these two entities.

Network analysis of 95 institutions (threshold ≥ 10 publications, Figure S3A) shows that international cross-border cooperation is relatively limited compared with domestic cooperation. It is worth noting that Chinese institutions (coded purple/red in the figure) have formed a domestic cooperation cluster centered on the CAS and Soochow University (Figure S3A). Timeline visualization (Figure S3B) shows that the intensity of this domestic collaboration has increased significantly since 2018, reflecting the formation of a strong regional research consortium within China.

### Contribution of authors

**Fig. 6:**
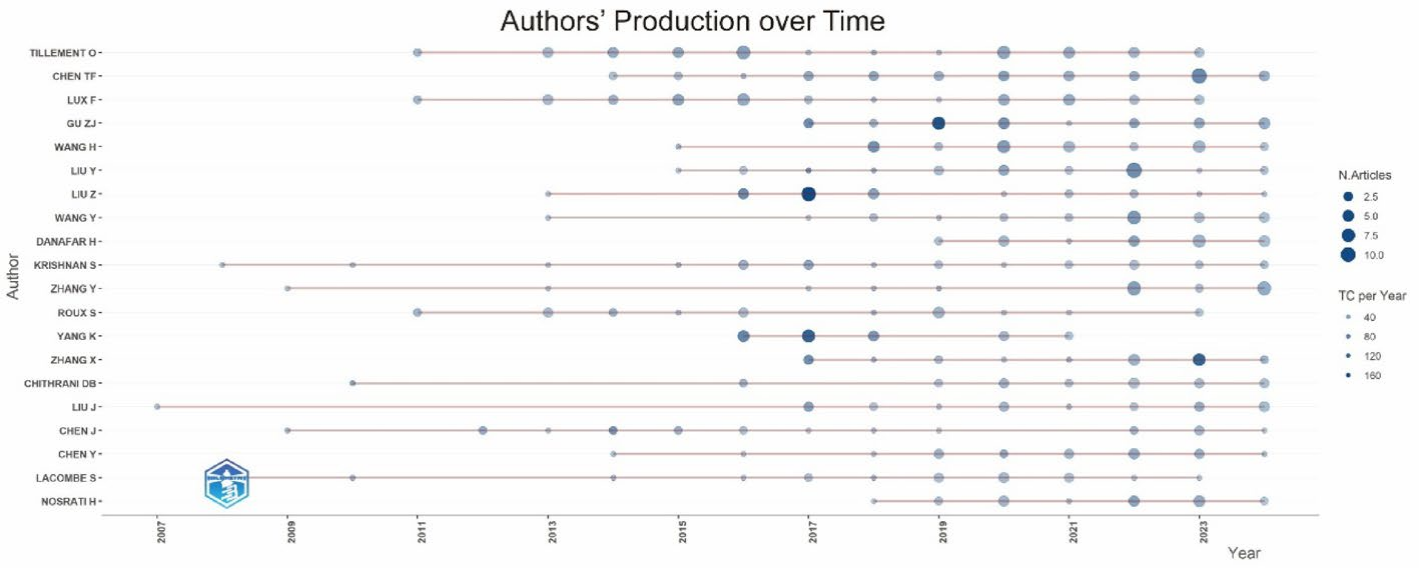
Top 20 authors’ temporal distribution (TC per Year: Total Citations per Year)

According to the H-index and publication counts, the leading authors in the field of nanoradiosensitizer research were determined. As shown in Figure S4A and Figure S4B, CHEN T.F. (Jinan University), LUX F. and TILLEMENT O. (Université Claude Bernard Lyon 1) achieved the highest H-index and publication counts. Figure 6 illustrates the temporal distribution of the publication output and academic impact of the major authors. Most of the high-yield authors have been active since 2010, and the number of publications has increased significantly since around 2015, indicating that the field has entered a rapid development stage (Fig. 6). Among the major contributors, authors such as TILLEMENT O., CHEN T.F. and LUX F. maintained consistent output, reflecting their long-term in-depth participation in this field (Fig. 6). In contrast, although KRISHNAN S. and ZHANG Y. entered the field earlier, their publications showed a certain degree of discontinuity (Fig. 6). Some authors have performed well in “TC per year” (darker colors in the figure), indicating that although their publication counts are relatively low or comparable to those of others, their research results have higher academic influence (Fig. 6).

The author’s cooperative cluster analysis (Figure S4C, threshold ≥ 8 publications) identified a total of 8 major collaborative groups. Among them, the French research team led by TILLEMENT O. and LUX F. constituted the largest collaboration network (Figure S4C), indicating their high level of collaborative capability and significant academic influence in this field. In contrast, research in China exhibits a “multi-center” pattern, with numerous independent collaboration clusters that outnumber those of other countries, (Figure S4C), reflecting a broad and diverse research base within China.

### Journals and co-cited journals

Table 1 lists the top 20 high-yield journals. The top 5 journals were *International Journal of Nanomedicine* (68 articles), *ACS Nano* (51), *ACS Applied Materials & Interfaces* (45), *Biomaterials* (45), and *International Journal of Molecular Sciences* (43). Notably, all the top 20 journals were located within JCR Q1 or Q2 categories, among which *Advanced Materials* held the highest impact factor (IF = 26.8), indicating the overall high quality of research in this domain.

**Table 1:** Top 21 most active journals in nanoradiosensitizer reseach

Ranking	Sources title	Output	% of 1866	IF 2024	JCR quartile 2024
1	INTERNATIONAL JOURNAL OF NANOMEDICINE	68	3.64	6.5	Q2/Q1/Q1/Q1
2	ACS NANO	51	2.73	16	Q1/Q1/Q1/Q1/Q1/Q1/Q1/Q1
3	ACS APPLIED MATERIALS & INTERFACES	45	2.41	8.2	Q1/Q1/Q1/Q1
4	BIOMATERIALS	45	2.41	12.9	Q1/Q1/Q1/Q1
5	INTERNATIONAL JOURNAL OF MOLECULAR SCIENCES	43	2.30	4.9	Q1/Q2/Q2/Q2
6	PHYSICS IN MEDICINE AND BIOLOGY	41	2.20	3.4	Q2/Q1/Q2/Q2
7	NANOSCALE	39	2.09	5.1	Q2/Q2/Q2/Q1/Q2/Q2/Q2/Q2
8	SCIENTIFIC REPORTS	35	1.88	3.9	Q1/Q1
9	MEDICAL PHYSICS	34	1.82	3.2	Q1/Q1
10	JOURNAL OF MATERIALS CHEMISTRY	33	1.77	5.7	Q2/Q2
11	NANOMATERIALS	32	1.71	4.3	Q2/Q2/Q2/Q2/Q2/Q2/Q2/Q2
12	JOURNAL OF NANOBIOTECHNOLOGY	31	1.66	12.6	Q1/Q1/Q1/Q1
13	PHARMACEUTICS	29	1.55	5.5	Q1/Q1
14	ADVANCED MATERIALS	28	1.50	26.8	Q1/Q1/Q1/Q1/Q1/Q1/Q1/Q1 /Q1/Q1/Q1/Q1
15	CANCERS	28	1.50	4.4	Q2/Q2
16	NANOMEDICINE-NANOTECHNOLOGY BIOLOGY AND MEDICINE	24	1.29	4.6	Q2/Q2/Q2/Q2
17	RADIATION PHYSICS AND CHEMISTRY	24	1.29	3.3	Q2/Q1/Q1/Q2/Q1/Q1
18	ADVANCED FUNCTIONAL MATERIALS	23	1.23	19	Q1/Q1/Q1/Q1/Q1/Q1/Q1/Q1 /Q1/Q1/Q1/Q1
19	ADVANCED HEALTHCARE MATERIALS	23	1.23	9.6	Q1/Q1/Q1/Q1/Q1/Q1
20	ADVANCED SCIENCE	22	1.18	19	Q1/Q1/Q1/Q1/Q1/Q1/Q1/Q1 /Q1/Q1/Q1/Q1
21	NANO TODAY	22	1.18	9.6	Q1/Q1/Q1/Q1/Q1/Q1

Impact factors and JCR quartile rankings were obtained from the 2024 Journal Citation Reports (Clarivate Analytics). Multiple quartile entries indicate that a journal is assigned to multiple Web of Science subject categories, and quartile rankings are reported for each corresponding category

Longitudinal analysis (Figure S5A) illustrates that the publication volume in these core journals remained low before 2012, and has accelerated significantly after 2018. Among them, International Journal of Nanomedicine exhibited the most dramatic recent growth, reflecting the increasing attention to the field of nanoradiosensitizers (Figure S5A). In terms of academic impact (Figure S5B), *Biomaterials* (H-index 37) and *ACS Nano* (H-index 36) lead the field as the most academically influential journals.

**Fig. 7:**
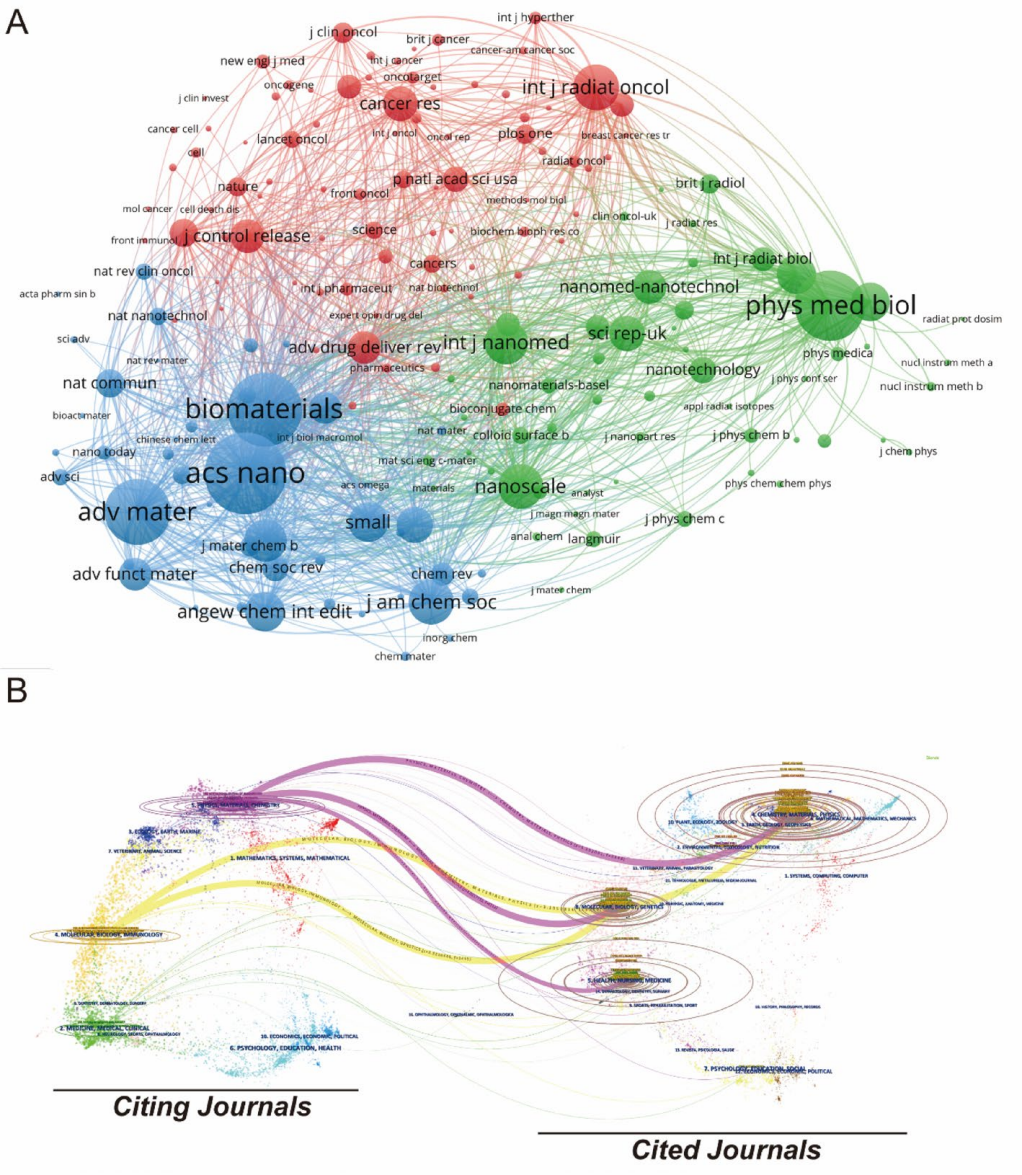
**A** Co-citation network. **B** Journal dual-map overlay

The journal co-citation network (Fig. 7A) revealed three main clusters, reflecting the different disciplinary foundations of nanoradiosensitizers. The “blue cluster” is mainly concentrated in the field of materials science and nanotechnology, including *ACS Nano*, *Advanced Materials* and *Biomaterials*, indicating the fundamental role of nanomaterial design in this field (Fig. 7A). The “green clustering” centers on physics-related journals, such as *Physics in Medicine and Biology* and *Nanomedicine: Nanotechnology, Biology and Medicine*, reflecting the cross-integration of physical dose enhancement mechanisms and biological effects (Fig. 7A). The “red cluster” consists of journals in tumor biology and clinical medicine, such as *International Journal of Radiation Oncology Biology Physics*, *Cancer Research* and *Journal of Controlled Release*, which reflects the importance of nanoradiosensitizers in clinical transformation and treatment applications (Fig. 7A). The three clusters are closely interconnected, highlighting the significant interdisciplinary and multidisciplinary characteristics of the field and the development trend from fundamental research to clinical translation.

The dual-map overlay analysis (Fig. 7B) illustrates the citation pathways between journals, revealing the characteristics of interdisciplinary knowledge flow in this field. Research outputs in this field are mainly published in journals of materials science, chemistry, and medicine (left side of the figure), whereas the cited literature predominantly originates from journals in physics, materials science, and molecular biology domains (Fig. 7B). This indicates that the field integrates multiple disciplines, including materials science, physics, and biomedicine, and exhibits a trend of evolving from a single physical mechanism toward a “physicobiological synergistic mechanism”. Notably, although journals in medicine, medical, and clinical fields have not yet formed independent citation pathways, emerging trends suggest an increasing interface between basic nanoscience and clinical applications, providing important opportunities for future translational research in this field (Fig. 7B).

## Keywords co-occurrence analysis

**Fig. 8:**
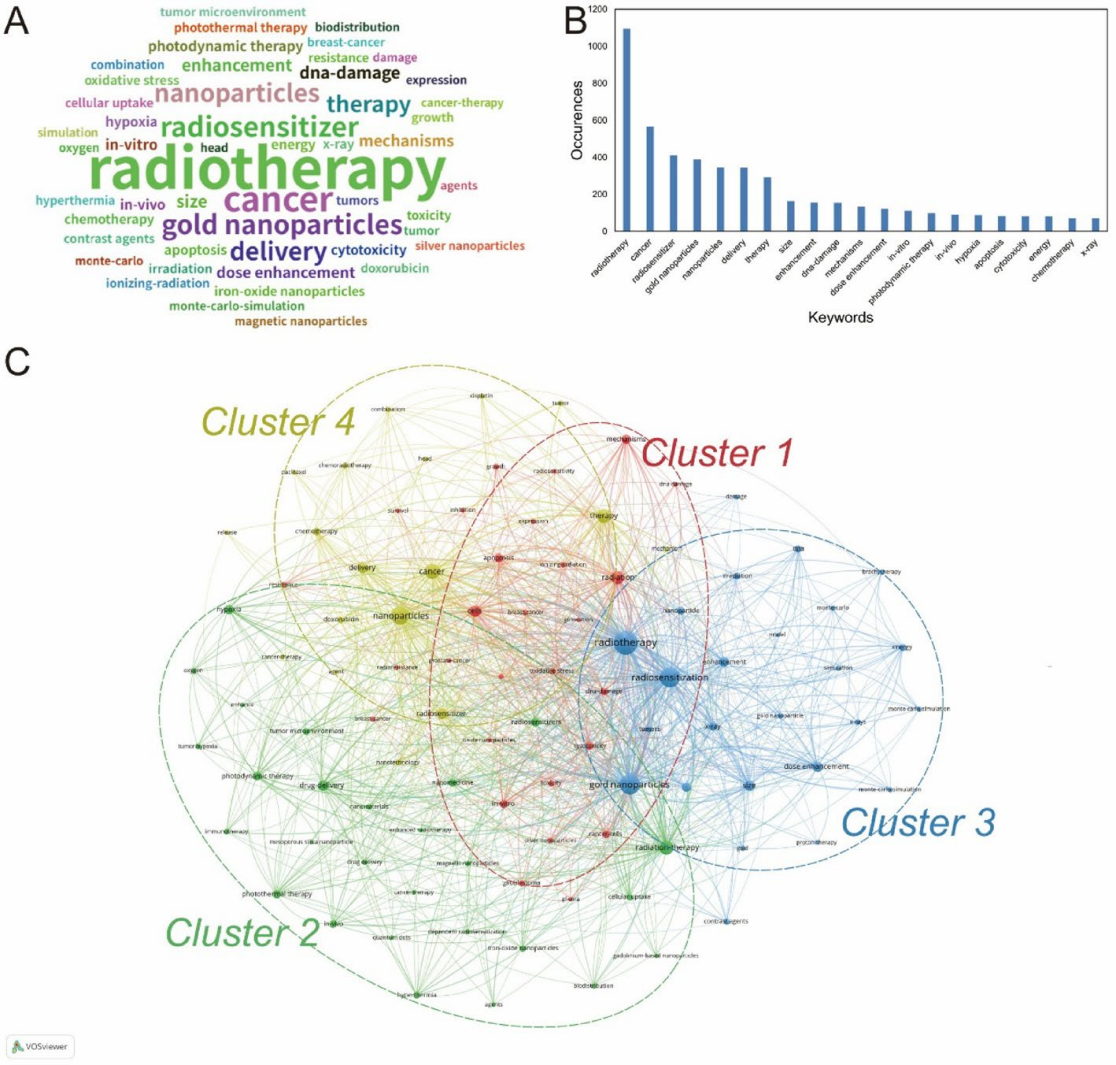
**A** Word cloud of keywords. **B** Top 20 keywords bar chart. **C** Network visualization map of keyword co-occurrence analysis. In this map, keywords with close relationships are grouped into the same cluster and represented by the same color. All keywords are categorized into four clusters: Cluster #1 (red nodes), Cluster #2 (green nodes), Cluster #3 (blue nodes), and Cluster #4 (yellow nodes)

Initial analysis using keyword word cloud (Fig. 8A) and frequency statistics (Fig. 8B) identified “radiotherapy”, “cancer”, “radiosensitizer”, “gold nanoparticles”, and “nanoparticles” as the five most frequent terms. Among them, “radiotherapy” occupied the most central and prominent position, indicating its core role in research (Fig. 8A, B). Furthermore, the emergence of emerging keywords such as “TME”, “DNA damage”, and “oxidative stress” suggests that research hotspots are gradually shifting toward biological mechanisms (Fig. 8A, B).

To decode the underlying thematic structure, a co-occurrence network was constructed (Fig. 8C, threshold ≥ 30 occurrences), which identified four research distinctions: Cluster #1 (red) primarily focuses on radiotherapy and its sensitization mechanisms; Cluster #2 (green) is associated with tumor biology and multimodal treatment strategies, including keywords such as “TME” and “photodynamic therapy (PDT)”; Cluster #3 (blue) mainly involves the physicochemical properties of nanomaterials, including “physical dose enhancement”, “X-rays”, etc.; Cluster #4 (yellow) represents interdisciplinary directions, involving combination therapy strategies and novel nanotechnology applications (Fig. 8C). These clusters also reveal the interdisciplinary and cross-disciplinary integration status of the field.

Temporal overlay analysis (Figure S5C) reveals a clear evolutionary trajectory: keywords in Cluster #3 emerged relatively early, which laid a physical foundation for the field; the keywords in Clusters #1, #2, and #4 appeared late, especially those related to biological mechanisms and immunotherapy, which represented the current research hotspots.

### Evolution of research hotspots

**Fig. 9:**
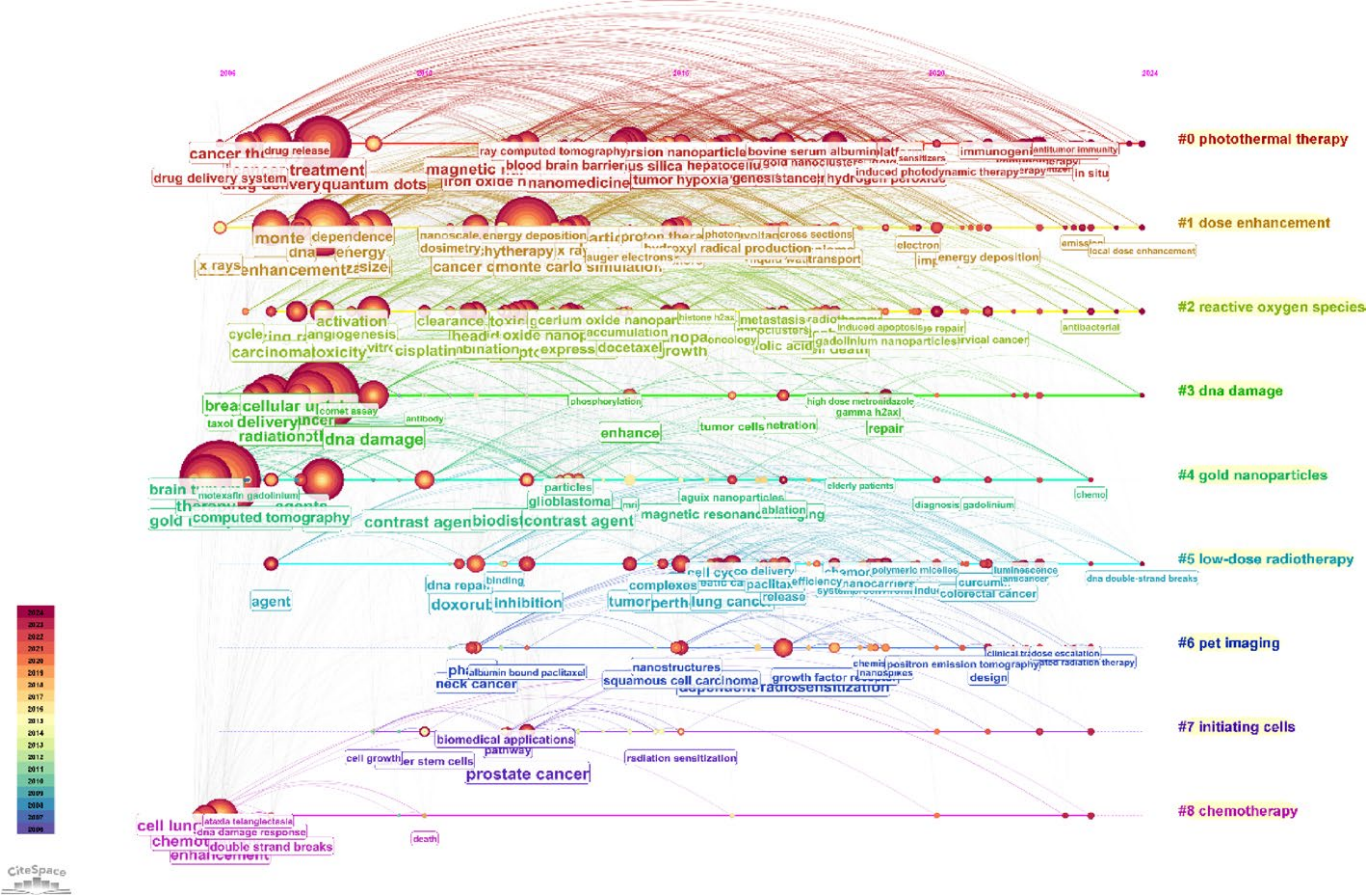
Keyword co-occurrence analysis. In this timeline view, the position of the node on the horizontal axis indicates the time point of the first occurrence, and the lines connecting the nodes represent co-occurrence relationships. The size of the node is proportional to the occurrence frequency of the keyword. The color gradient from purple to red corresponds to the period from 2006 to 2024 as shown in the color bar

The timezone view (Fig. 9) visualizes the temporal evolution of hotspots (Modularity Q = 0.3536, Mean Silhouette S = 0.6591), confirming a robust network structure. Keywords such as “dose enhancement,” “ROS,” “DNA damage,” and “low-dose radiotherapy” have remained prominent through 2024, underscoring their enduring relevance (Fig. 9).

**Fig. 10:**
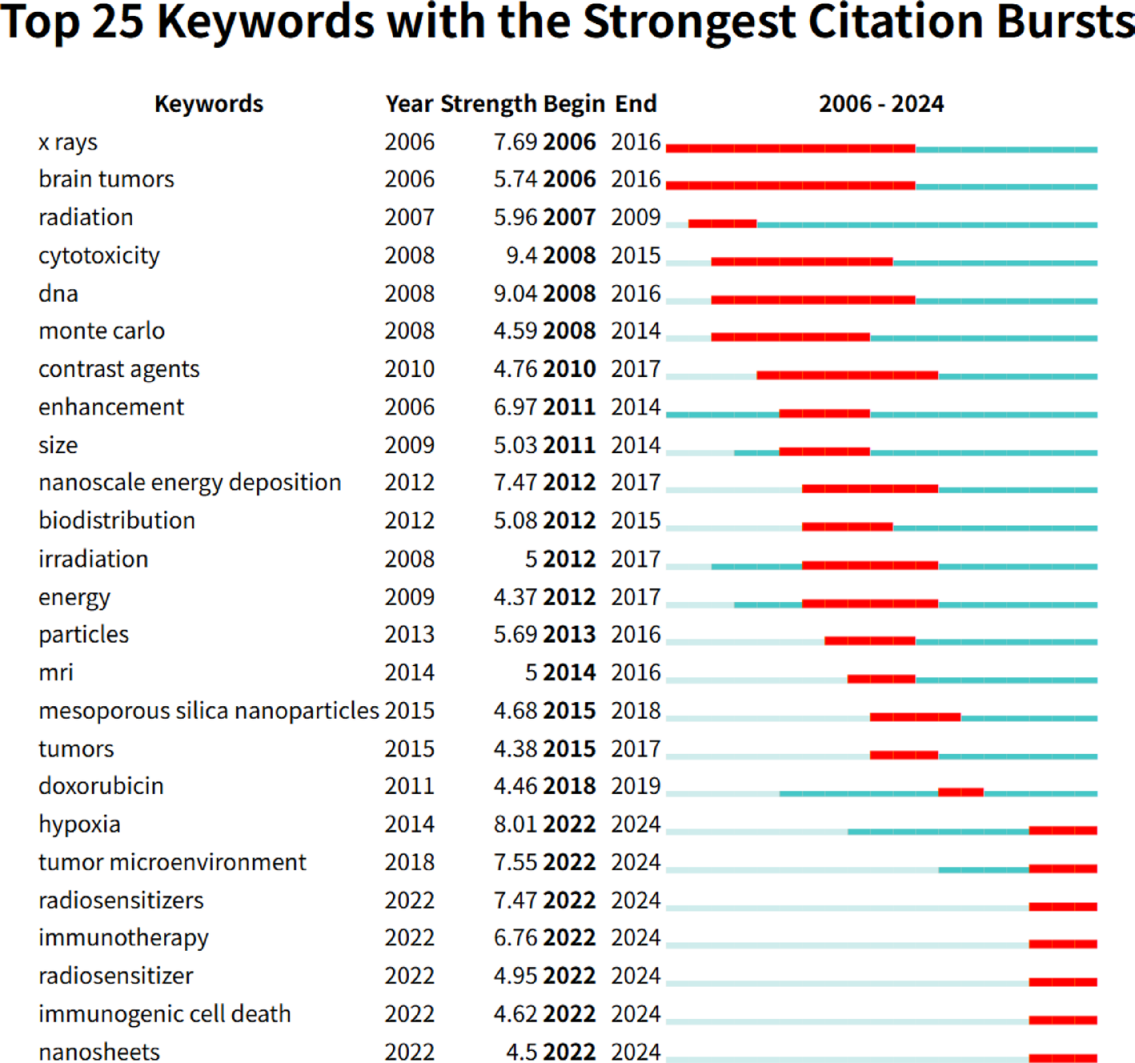
Top 25 keywords with the strongest citation bursts by CiteSpace. A blue line indicates the timeline, and the bars in red stand for a burst period

Keyword burst detection (Fig. 10) displays the top 25 keywords with the highest burst strength. We categorized keywords that remained active until 2024 into three frontier directions: (1) Biological Mechanisms, keywords like “hypoxia” (Strength: 8.01), “TME” (7.55), and “immunogenic cell death (ICD)” (4.62), indicating attention to biological modulation. (2) Therapeutic strategies, the strong burst of “immunotherapy” (6.76) and “sonodynamic therapy” (3.20) reflects the community's intense focus on integrating radiotherapy with systemic immune activation and novel dynamic therapies. (3) Materials innovation, terms like “nanosheets” (4.50), “design” (3.49) and “strategy” (4.01) highlight the continuous refinement of nanomaterial architecture for optimized delivery and efficacy (Fig. 10). Collectively, these bursts keywords indicated that recent studies have increasingly focused on biological regulation and immunotherapy.

### Analysis of references and co-cited references

**Fig. 11:**
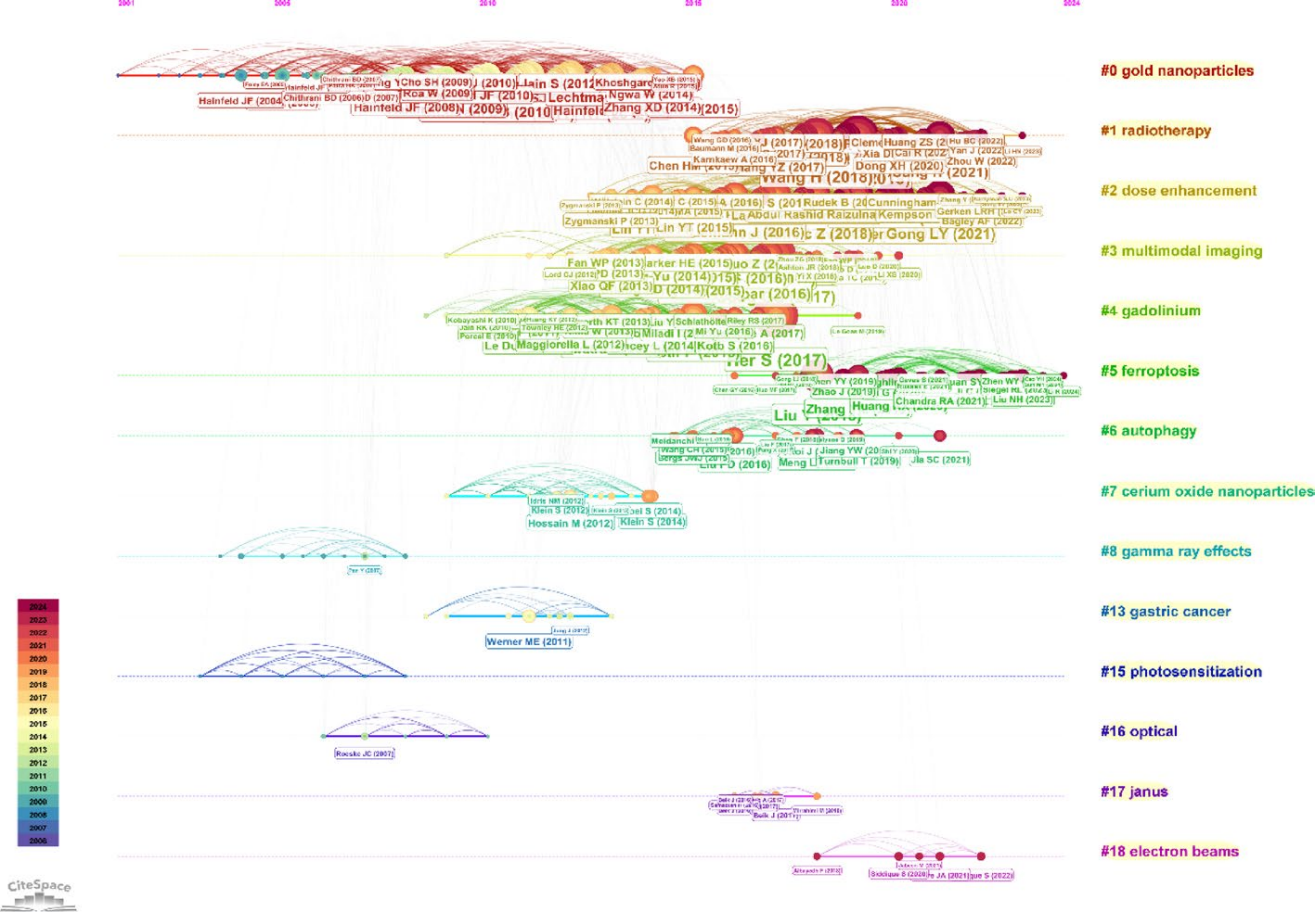
Reference co-citation analysis. In this timeline view, the position of each node on the horizontal axis indicates the time point of its first appearance, and the lines connecting nodes represent co-citation relationships. The size of a node is proportional to the number of citations of the corresponding reference. A color gradient from purple to red is used, corresponding to the period from 2006 to 2024 as indicated in the color bar

References cluster analysis (Fig. 11) identified 19 distinct thematic cluster (Modularity Q = 0.673, Silhouette S = 0.8622), indicating a well-structured and highly reliable knowledge network. The analysis reveals a clear evolution of research focus within the field.

Early research hotspots were primarily centered on fundamental material exploration and radiation effects, represented by clusters such as “Gold nanoparticles” (Cluster #0) and “Gamma ray effects” (Cluster #8) (Fig. 11). In contrast, more recent studies have shifted toward clinically relevant and mechanistically driven topics, including “Radiotherapy” (Cluster #1), “Dose enhancement” (Cluster #2), and notably “Ferroptosis” (Cluster #5) (Fig. 11). The emergence of ferroptosis as a distinct cluster highlights a novel mechanistic intersection between nanomedicine and radiation biology, reflecting a transition from material-based studies to pathway-oriented therapeutic strategies.

**Fig. 12:**
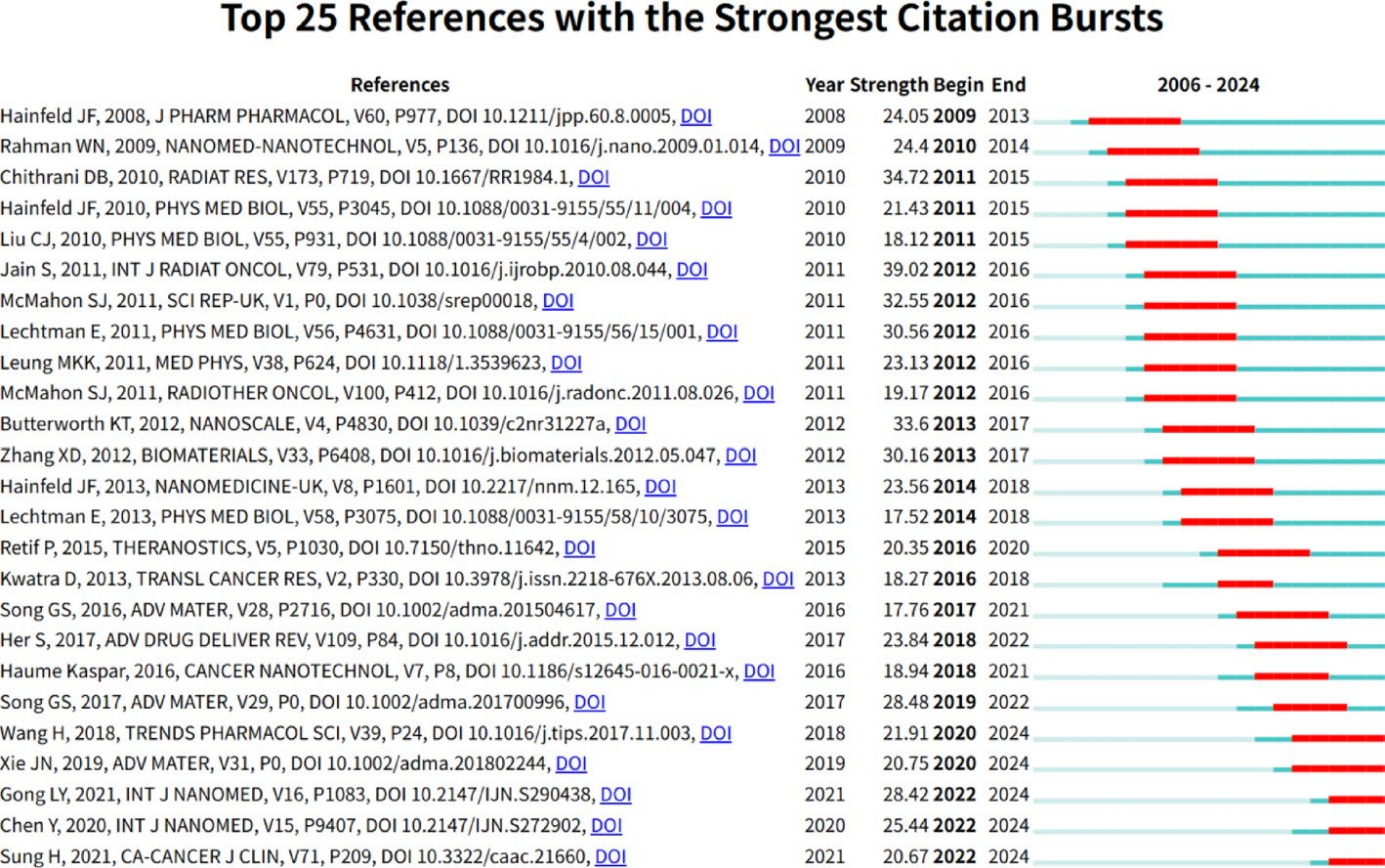
Top 25 references with the strongest citation bursts by CiteSpace. A blue line indicates the timeline, and the red bars stand for the burst period of each reference

Furthermore, reference burst analysis (Fig. 12) covers the period from 2006 to 2022 and highlights a series of influential works associated with these evolving themes. Collectively, these findings suggest that research attention has gradually expanded from material development to studies involving biological mechanisms and potential clinical applications.

## Discussion

### Global research landscape

Our bibliometric analysis has revealed that in the past two decades, the field of nanoradiosensitizers has shown a three-stage growth pattern: a slow growth stage (2006 ~ 2011), a rapid expansion stage (2012 ~ 2022), and a high-yield stable stage (2023 ~ 2024) (Fig. 2). The year 2012 marked a critical turning point in the field's development, where breakthroughs in nanomaterial design and functionalization strategies led to the development of a large number of gold-based nanoradiosensitizers [39–41], thereby propelling the field into a phase of rapid growth. The cumulative publications in the four years from 2021 to 2024 alone reached 894 (47.9% of the total), highlighting the vitality of this field (Fig. 2). As shown in Figure S1, the annual citation frequency showed a synchronous upward trend, reaching a peak (9487 times) in 2017, reflecting the increasing academic influence in this field. These trends indicate that nanoradiosensitizers have become a research hotspot at the intersection of cancer therapy and nanomaterials.

Geographically, China and the United States are undoubtedly the core areas of research in this domain (Fig. 4). The formation of this landscape is closely related to the substantial and sustained investments made by both governments in the field of nanomaterials science. However, as shown in our institutional network analysis (Figure S2), current international cooperation remains fragmented. Therefore, the establishment of bridges through cross-border cooperation is crucial for standardizing protocols and accelerating clinical translation.

### Evolution of mechanisms

#### From physical radiosensitization to biological functional regulation

Tracing the developmental trajectory of the field suggests a clear transition in the underlying scientific paradigms. In 1998, Regulla et al. theoretically demonstrated that high-Z materials can significantly amplify localized radiation doses by triggering the photoelectric effect and generating secondary electrons, such as Auger electrons [42]. This discovery laid the conceptual foundation for the field of nanoradiosensitization. Over the following decade, research in this field primarily focused on a “material-centered” design paradigm, aiming to optimize the physicochemical properties of high-Z nanoparticles (such as Au, Pt, and Bi) to maximize the physical dose enhancement factor (DEF) [42, 43]. This approach sought to increase energy deposition within tumors while minimizing radiation damage to surrounding healthy tissues.

However, classical physical radiosensitization models could not fully account for the complex biological responses of nanomaterials in vivo. Until 2008, Ni et al. reported that the radiosensitizing effect of fullerene-C60 (nano-C60) derivatives did not arise from physical energy absorption, but from the induction of intracellular ROS generation and oxidative damage to cell membrane structures [44]. This discovery revealed the unique catalytic and biological activities of nanomaterials in radiation fields, marking a critical transition in nanoradiosensitizer research from “technical parameter optimization” to “biological function regulation”.

Keyword evolution analysis confirms this trend. Early studies (represented by blue nodes in Cluster #3) were primarily concentrated on material development and the establishment of technical methodologies (Fig. 8C, S5C). As the research in this field enhances, the research emphasis progressively shifted toward more in-depth mechanistic exploration (Cluster #1) and advanced directions such as combination therapy strategies (Clusters #2 and #4) (Fig. 8C, S5C). This color distribution of the clusters suggests that research attention has gradually shifted from material development to studies focusing on biological mechanisms and translational applications (Fig. 9). The recent hot topics have shifted from single “gold nanoparticles” (Cluster #4) to complex biological mechanisms such as “reactive oxygen species” (Cluster #2), “ferroptosis” (Cluster #5), and “autophagy” (Cluster #6) (Fig. 11). These findings suggest that recent studies have paid more attention to the biological effects of nanomaterials during radiotherapy. Current research mainly focuses on two major research themes: amplifying oxidative stress (such as Fenton or Fenton-like reaction-induced ferroptosis [45–47]) and remodeling the TME (such as alleviating hypoxia [48]). Compared with traditional physical radiosensitization strategies, these studies increasingly explore biological mechanisms that may influence radiotherapy response.

#### Amplifying oxidative stress

When ionizing radiation interacts with biological tissues, it primarily triggers the radiolysis of water, leading to the generation of various ROS [49]. Among these, the hydroxyl radical (·OH) is recognized as the most destructive factor due to its exceptional reactivity in inducing oxidative damage to biomacromolecules [50].

To amplify this effect, transition metal-based nanozymes (e.g., Fe, Cu, or Mn-based nanosystems) have been developed to catalyze in situ Fenton or Fenton-like reactions [45–47]. By converting endogenous hydrogen peroxide (H_2_O_2_) into ·OH radicals, these nanozymes effectively bypass the oxygen-dependent limitations of traditional radiotherapy. This surge in radical production not only exacerbates direct DNA damage but also promotes sustained lipid peroxidation, leading to the emergence of ferroptosis-mediated sensitization as a major research hotspot [51–53].

For instance, Liu et al. engineered oxygen vacancy-rich MnO_2_ nanoflowers (ovs-MnO_2_), which acts as a Fenton-like reaction activator [54]. This system enhances intracellular ROS accumulation and further improves radiotherapy efficacy by triggering ferroptosis [54]. Similarly, Gu et al. developed biomimetic Cu_2_WS_4_ (CWP) nanozyme with multiple enzyme-like activities [47]. CWP exhibits strong Fenton-like catalytic performance, generating abundant ROS and inducing ferroptosis. By integrating both physical radiosensitization and biological catalytic effects, it significantly enhances the therapeutic efficiency of radiotherapy in breast cancer [47].

#### Remodeling the TME

The TME is characterized by chronic hypoxia, a highly reductive state (e.g., elevated GSH levels), and an immunosuppressive landscape, all of which severely compromise RT sensitivity [55–57].

To address hypoxia within the TME, various nanoradiosensitizers have been developed to catalyze the decomposition of H_2_O_2_ or to deliver and release O_2_ in situ, thereby achieving localized oxygen supply. This strategy enhances the oxygen fixation effect and has emerged as a key chemical radiosensitization approach. For example, Xu et al. designed an ozone-loaded platinum (IV) prodrug nanosystem (Pt(IV)-PFOA) [58]. In this platform, the O_2_ generated from ozone decomposition effectively alleviates tumor hypoxia. Moreover, ozone produces ROS under X-ray irradiation, disrupting intracellular redox homeostasis, triggering a GSH-mediated reductive response that facilitates the controlled conversion of the Pt (IV) prodrug into its active Pt (II) form. More importantly, the synergy between radiotherapy and ozone generates an amplified surge of ROS within the tumor, which significantly triggers a robust ICD burst. This process promotes the release of tumor-specific neoantigens, successfully reshaping the TME into an immuno-favorable state while minimizing systemic toxicity [58].

Beyond oxygen supply, the disruption of TME redox homeostasis represents another frontier. To synergistically sensitize tumor cells to radiotherapy, Qiao et al. developed a high-performance heterojunction nanosystem (Se@PB) designed to disrupt the redox homeostasis of the TME [59]. By doping Se into Prussian blue (PB) nanoparticles with mixed-valence (Fe^2+^/Fe^3+^), an Fe^2+^-Se-Fe^3+^ electron transfer chain was successfully constructed. This chain significantly amplified the X-ray-induced photocurrent effect and facilitated high-energy electron transport, thereby enhancing physical radiosensitization. Concurrently, Se introduction elevated the Fe^2+^/Fe^3+^ ratio and promoted the oxidation of Se to the Se^4+^ state. This conversion process effectively depleted intracellular GSH and perturbed the TME redox balance, ultimately bolstering biochemical radiosensitization [59].

These representative studies collectively highlight that the current research frontier is characterized by the synergistic integration of advanced material chemistry and molecular cell biology. This transition from simple dose deposition to complicate microenvironmental molecular regulation marks one of the most profound paradigm evolutions in the field over the past two decades.

### Tackling radioresistance: emerging strategies and therapeutic opportunities

Tumor radioresistance arises from multiple mechanisms, mainly including enhanced cellular DNA repair capacity, the protective effects of the TME, and the presence of cancer stem cells (CSCs) [60]. These intrinsic and acquired mechanisms pose a major barrier to clinical translation. Our analysis has identified three technological breakthrough strategies that could help overcome these barriers:

#### Targeting CSCs

CSCs, owing to their robust self-renewal capacity and efficient DNA repair mechanisms, are a key driver of tumor recurrence and metastasis after radiotherapy [61].

To address this clinical challenge, the development of CSCs-targeted nanoradiosensitization systems has become a major research focus. One highly regarded strategy involves using cell membranes to create biomimetic nanomaterials, thereby endowing them with CSCs-targeting capabilities [62, 63]. By recognizing CSCs-associated surface markers (e.g., CD41), these platforms can achieve more precise delivery of ROS-generating payloads, effectively eradicating the radioresistant seed cells [62]. For instance, Ning et al. developed a biomimetic nanosystem (PMT) by loading the Type I aggregation-induced emission (AIE) photosensitizer TBP-2 into organic mesoporous silica nanoparticles encapsulated with platelet membranes [63]. This system leverages disulfide bonds to achieve responsive degradation and TBP-2 release within the high GSH levels in the TME. Upon light excitation, TBP-2 triggers Type I PDT, generating a surge of ·OH that simultaneously depletes GSH and induces severe DNA damage in CSCs. Results confirmed that, when combined with radiotherapy, this system could completely eradicate CSCs without observed tumor recurrence [63].

Beyond biomimetic delivery for direct eradication, the delivery of functional molecules to modulate the CSC stemness phenotype represents another potent strategy for synergistic radiosensitization. Zhou et al. designed a multifunctional nanoplatform based on bismuth sulfide nanoflowers (Bi@PP) to encapsulate the CSC regulator miR-339-5p [64]. In this system, miR-339-5p effectively reverses CSCs stemness and overcomes radiation resistance by targeting the ubiquitin-specific peptidase 8 (USP8) pathway. Concurrently, the high K-edge value and superior X-ray attenuation coefficient of bismuth sulfide enable CT imaging-guided precision radiosensitization [64].

To alleviate the hypoxic microenvironment in deep tumors and suppress CSCs stemness, Chen et al. employed a natural fatty acid eutectic mixture as a phase-change material (PCM) to encapsulate thermos-responsive micelles, pre-oxygenated perfluoropentane (an O_2_ carrier), and the sensitizer metronidazole (MTZ) [65]. Under 808 nm near-infrared (NIR) irradiation, the PCM acts as a temperature-sensitive "gatekeeper," undergoing a solid-to-liquid phase transition to precisely control the release of O_2_ and MTZ. This design not only enhances extracellular matrix (ECM) permeability but also effectively reverses hypoxia-induced CSCs radioresistance in triple-negative breast cancer (TNBC), thereby significantly augmenting the overall efficacy of radiotherapy [65].

#### Optimizing delivery for precision radiotherapy

Analysis of reference cluster #18 (electron beams) indicates that the current research is highly focused on integrating nanoradiosensitizers with advanced radiotherapy modalities (Fig. 11). Electron beams, characterized by their dose concentration in superficial tissues, are widely used in treating superficial tumors such as skin cancer. Monte Carlo simulation studies have systematically evaluated the radiosensitization performance of various nanoparticles under electron beams of different energies, providing a theoretical foundation for optimizing combined electron beam radiotherapy with nanosensitization strategies for superficial tumors [66–68].

For deep tumors, existing research places greater emphasis on proton therapy. The unique physical characteristic of proton beams—the Bragg peak—enables precise energy deposition within deep-seated tumor targets while maximally protecting surrounding normal tissues. By establishing a treatment model that combines precise proton energy deposition with synergistic enhancement by nanoradiosensitizers, the efficacy of radiotherapy for deep-seated tumors can be significantly improved [69].

For example, PEG-modified boron nanoparticles [70, 71] and gold nanoparticles [69] have demonstrated excellent sensitization potential within this combined strategy, emerging as key research directions in precision radiotherapy for deep-seated tumors.

#### Real-time monitoring and theranostics

The emergence of keyword cluster #6 (PET imaging) provides technical support for addressing the challenge of evaluating radiotherapy resistance (Fig. 9). Researchers have developed theranostic nanoplatforms by integrating nanoradiosensitizers with PET imaging agents (such as ⁶^4^Cu^2^⁺). These platforms enable real-time, non-invasive monitoring of the accumulation and distribution of sensitizers at tumor sites, thereby informing the development of personalized radiotherapy protocols (e.g., adjusting dosage and irradiation timing) [72, 73].

For example, Shen et al. (2017) developed ultrasmall coordination polymer nanodots (CPNs) based on tungsten ions (W^6+^) and gallic acid (GA), and prepared W-GA-PEG CPNs through PEG modification [72]. These CPNs can not only be efficiently labeled with ⁶^4^Cu^2^⁺ for PET imaging, clearly demonstrating their efficient tumor accumulation and in vivo metabolism, but also exhibit significant radiosensitizing effects due to the high X-ray absorption capacity of tungsten, effectively inhibiting tumor growth [72]. Such multimodal nanoplatforms pave a new technological path for achieving precise and personalized radiotherapy, holding promising clinical translation prospects.

### The translational hurdle

The long-term toxicity, organ accumulation, and immunogenicity of nanomaterials represent formidable challenges in their transition from bench to bedside. Current research is addressing these challenges through breakthroughs in both material design and safety evaluation standards.

In material design, the research focus has shifted toward developing biodegradable/clearable nanosensitizers, such as those based on natural polymers (e.g., chitosan, sodium alginate), MOFs, or peptide-based degradable materials. For instance, Chen et al. (2021) synthesized PEGylated Fe_3_O_4_ magnetic nanoparticles, which demonstrated favorable biosafety profiles in both hematological indices and histological examinations [74].

Regarding safety evaluation standards, the research paradigm has evolved from focusing primarily on acute toxicity to emphasizing systematic assessment of long-term, multi-organ, and immunotoxicity. For example, utilizing animal models that more closely mimic human physiology to evaluate the effects of long-term (e.g., 6–12 months) nanomaterial accumulation on the function and histology of critical organs like the liver and kidneys [75]. Employing organoid models (e.g., tumor organoids, normal tissue organoids) to precisely assess the “tumor-targeting toxicity” of nanoparticles, ensuring any damage to normal tissues remains within clinically acceptable limits [2, 76].

Despite the growing body of research on nanoradiosensitizers, the field remains in its early stages. In-depth exploration of their biosafety is still insufficient, and clinical translation faces numerous challenges: the complex design of current nanosystems carries high potential safety risks, hindering large-scale clinical application; furthermore, the long-term efficacy, biocompatibility, and biodegradability of nanomaterials require further validation and optimization.

### Future frontiers

Burst detection analysis provides useful insights into changes in research attention over time (Fig. 5B). Our results show that the keywords with sustained burst strength through 2024 have gradually shifted from broad concepts to more specific topics, with particular attention to immunotherapy, ICD, and nanosheets (Fig. 9). These trends suggest increasing interest in the biological mechanisms and potential applications of nanoradiosensitizers.

#### The era of radio-immunotherapy synergy

The integration of nanoradiosensitizers with immunotherapy represents one of the most actively investigated research directions [77–79]. Conventional immunotherapy, particularly immune checkpoint blockade, is often hindered by an immunosuppressive TME, limited antigen presentation, and insufficient infiltration of effector immune cells. In contrast, nanoradiosensitizers not only directly enhance tumor cell killing during radiotherapy but also improve the response rate to immunotherapy by modulating the immune microenvironment (e.g., promoting IFN-β release, activating DCs and T cells) [48].

##### Amplifying ICD

Radiotherapy itself can induce ICD in tumor cells, leading to the release of damage-associated molecular patterns (DAMPs), such as ATP, HMGB1, calreticulin, and subsequently activating the adaptive immune response [79–81]. While ionizing radiation itself can trigger the release of DAMPs, the integration of nanoradiosensitizers significantly amplifies this process by exacerbating cellular oxidative stress and endoplasmic reticulum (ER) stress. These molecular signals collectively transform the irradiated tumor into an “in situ vaccine”, effectively promoting the recruitment and maturation of DCs and subsequently priming a robust, adaptive anti-tumor T-cell response [81]. Currently, the “molecular mechanisms by which nanoradiosensitizers regulate ICD” have become a key research focus.

For instance, Zheng et al. engineered a mesoporous organosilicon (HMON)-based nanoplatform (PPI-AnnV@MMC@HA) with a hybrid –Si–O–M– framework via Mn and Ca doping [82]. This system was co-loaded with Omeprazole (PPI) and the efferocytosis inhibitor AnnV, and further functionalized with hyaluronic acid (HA) for active tumor targeting. Mechanistically, Ca^2^⁺ induces mitochondrial overload, while Mn^2^⁺ triggers a burst of ROS through Fenton-like reactions. This ion-interference amplification strategy not only enhances radiotherapy efficacy but also significantly amplifies ICD and activates the cGAS–STING signaling pathway, thereby promoting systemic anti-tumor immune responses [82].

In parallel, remodeling of the hypoxic and immunosuppressive TME has emerged as another effective strategy for potentiating ICD. Qian et al. constructed a PEGylated core–shell nanoparticle (GMCN@PEG) by depositing MnO₂ onto gold nanoparticles (GNP) [83]. The MnO₂ shell catalyzes the decomposition of H₂O₂ into oxygen, alleviating tumor hypoxia, while exhibiting peroxidase (POD)-like activity to generate ROS. Meanwhile, the GNP core enhances radiation energy deposition, leading to increased DNA double-strand breaks (DSBs). This synergistic effect promotes ICD, activates the cGAS–STING pathway, and reprograms the immunosuppressive tumor microenvironment, ultimately enhancing radiation-induced anti-tumor immunity [83].

##### Enhancing immune checkpoint blockade (ICB)

The clinical efficacy of ICB is often hindered by the low response rates of “cold tumors” and the risk of systemic immune-related adverse events [84]. Recent research has shifted toward optimizing combination strategies of “nanoradiosensitizers—immune checkpoint inhibitors” to achieve localized immune activation.

One important direction involves gene therapy-based regulation of immune checkpoints [85, 86]. For example, Wang et al. developed an Au/MnO₂-based nanoplatform for the delivery of programmed death-ligand 1 (PD-L1) siRNA into tumor cells [86]. This nanoradiosensitizer leverages the physical properties of high-Z elements to enhance X-ray absorption while simultaneously utilizing the enzyme-like activity of MnO_2_ to alleviate tumor hypoxia. At the molecular level, it effectively downregulates PD-L1 expression via RNA interference (RNAi), thereby integrating gene regulation with radiotherapy. Through the combination of enhanced radiosensitization and in-situ immune activation, this system achieves a synergistic anti-tumor effect. Importantly, it promotes durable anti-tumor immune memory while significantly reducing systemic immune-related adverse effects, demonstrating the considerable advantages of this integrated therapeutic strategy [86].

Beyond gene silencing approaches, targeted blockade combined with imaging-guided nanoplatforms has also emerged as a promising strategy. Sun et al. constructed a Gd- hybridized Pt nanoplatform (GPPZ) incorporating a dimeric PD-L1 antagonistic affibody (ZPD-L1) [79]. The platinum component endows the system with catalase (CAT)- and POD-like activities, promoting the generation of O₂ and ·OH, thereby enhancing radiosensitivity and amplifying ICD. Meanwhile, ZPD-L1 effectively blocks the PD-1/PD-L1 interaction, reversing T cell immunosuppression and further enhancing anti-tumor immunity. In addition, this platform enables MRI contrast enhancement in tumors with high PD-L1 expression, demonstrating significant potential for theranostic applications [79].

Moreover, to pursue comprehensive systemic therapeutic outcomes, Tan et al. developed a biodegradable high-Z nanoradiosensitizer (Pt@Bi₂Se₃-RGD) and combined it with PD-L1 blockade therapy [87]. This strategy not only enhanced radiosensitization but also effectively suppressed both primary and distant tumors, while enabling photoacoustic imaging-guided radio-immunotherapy.

#### Next-generation platforms: the emergence of 2-dimensional (2D) nanosheets

Nanosheets, as an important class of 2D nanomaterials, demonstrate significant advantages in the design of radiosensitizers due to their unique structural properties [88–90]. There ultrahigh surface area and unique 2D structure facilitates enhanced interaction with tumor cell membranes, significantly improving cellular uptake efficiency [91]. Current research primarily focuses on the integration of nanosheets into multimodal combination therapies.

For instance, Shi et al. developed lanthanide-doped ultrathin Bi_2_O_2_CO_3_ nanosheets that leverage the radiotherapeutic properties of their high-Z elements (Bi, Yb, and Er), combined with the material's upconversion luminescence, to achieve a synergistic effect between radiotherapy and PDT [90].

For multifunctional composite nanoplatforms, Zhu et al. engineered a Ti_3_C_2_-MXene-based 2D nanosheet system (Au-Pt@Mxene@PVA) [92]. This platform consists of Ti_3_C_2_-MXene nanosheets as the core, coated with gold nanorods and a cisplatin shell, and further functionalized with polyvinyl alcohol (PVA). Benefiting from the excellent electronic properties and photothermal effect of Ti_3_C_2_-MXene, Au-Pt@Mxene@PVA enhances radiation-induced DNA damage and inhibits DNA damage repair mechanisms. In vitro studies demonstrated that this system increased ROS generation by approximately 2.6-fold compared to radiotherapy alone, thereby significantly improving radiosensitivity. Moreover, when combined with photothermal therapy (PTT), this platform enables effective tumor ablation through localized hyperthermia and helps overcome radioresistance in glioblastoma [92].

In addition, to address limitations of the TME, such as lactate accumulation, hypoxia, and insufficient ROS levels, Yao et al. designed a CoMnFe layered double oxide (LDO) nanosheet with multi-enzyme-like activities [93]. This nanozyme system catalyzes the conversion of H_2_O_2_ into O_2_ and ROS, thereby amplifying oxidative stress during radiotherapy**.** Simultaneously, it effectively depletes lactate, disrupting DNA and protein repair processes within tumor cells. Through this multi-pronged mechanism, the platform synergistically enhances radiotherapeutic efficacy and provides a novel strategy for modulating tumor metabolism and radiosensitivity [93].

Overall, nanosheets are driving the evolution of radiosensitization strategies from conventional single-function materials toward integrated, structure–function unified multimodal therapeutic platforms. However, with the emergence and development of these novel morphologies, future research must rigorously elucidate and address their long-term in vivo behavior and biosafety to bridge the gap between the novelty of material science and clinical applicability.

### Limitations

Although this study provides a series of informative findings, several limitations should be acknowledged.

First, the literature data analyzed in this study were exclusively sourced from the WoSCC database and did not include publications from other specialized databases such as PubMed, Embase, or Cochrane Library. Nevertheless, WoSCC is widely recognized as one of the most representative databases for bibliometric analysis, and its coverage is generally considered sufficient to reflect the overall development of a research field [28, 29]. In addition, constraints such as file format compatibility have blocked most bibliometric studies from adopting a multi-database merging approach [94–96]. Second, given the diversity of terminology used in studies related to nanoradiosensitizers, it is possible that the retrieval failed to include some relevant publications, despite the use of logical operators (e.g., “AND”, “OR”, “NOT”) in the search strategy to improve recall and precision.

Third, although institutional names were manually checked and standardized where necessary, residual uncertainties may remain due to variations in author name abbreviations, transliteration differences, institutional restructuring, and organization-level aggregation within the WoSCC database. For example, some institution-level entities indexed by WoSCC may represent integrated organizational systems rather than individual subunits. Likewise, ambiguous author names may not always be fully distinguishable. Therefore, institution- and author-level findings should be interpreted with appropriate caution.

Furthermore, some recently published high-quality studies may not have been included in the analysis due to their low citation frequency at the time of data collection. Therefore, as the literature in this field continues to grow, the identified research trends and hotspots in nanoradiosensitizers may evolve. Future studies could adopt more comprehensive data sources and finer-grained analytical methods to further validate and update these findings.

## Conclusions

This study provides a comprehensive bibliometric overview of nanotechnology in cancer radiosensitization and summarizes the global research landscape and developmental trends in this field. Our findings confirm that the field has transitioned from an exploratory phase into a period of exponential growth, driven by the core drivers from China and the United States. However, the fragmented nature of international collaboration highlights an urgent need for cross-border consortia to standardize protocols and accelerate progress.

The field is currently undergoing a profound paradigm shift: Mechanistically, there is a shift from simple physical dose enhancement toward precise biological modulation (e.g., alleviating hypoxia, inducing ferroptosis, etc.). Therapeutically, the field is advancing from monotherapy to synergistic multimodal therapies, with “radiotherapy–immunotherapy” and ICD representing the most promising frontiers. Materially, the focus is turning toward biodegradable, TME-responsive designs (e.g., nanosheets) to address long-standing biosafety concerns.

More critically, the next decade will be decisively defined as a period of "critical leap in translation." Future research should not only prioritize the synthesis of new materials but also dedicate efforts to the optimization of designs, rigorous evaluation of long-term safety, and the seamless integration with clinical radiotherapy workflows. By bridging the gap between material science innovation and clinical oncology needs, nanoradiosensitizers hold the potential to revolutionize cancer treatment paradigms. This study provides valuable references for researchers, and offers important insights for policymakers in decision-making processes.

## Supplementary Information

Below is the link to the electronic supplementary material.

Supplementary Material 1

## Data Availability

The datasets analyzed during the current study were obtained from the Web of Science Core Collection database. Further processed data are available from the corresponding author upon reasonable request.

## Abbreviations

AAY: Average appearance year
AIE: Type I aggregation-induced emission
CSCs: Cancer stem cells
CAT: Catalase
CNRS: Centre national de la recherche scientifique
DAMPs: Damage-associated molecular patterns
DEF: Dose enhancement factor
DSBs: DNA double-strand breaks
ECM: Enhances extracellular matrix
ER: Endoplasmic reticulum
HA: Hyaluronic acid
high-Z: High atomic number
H₂O₂: Hydrogen peroxide
OH: Hydroxyl radicals
ICB: Immune checkpoint blockade
ICD: Immunogenic cell death
LLC: Lewis lung carcinoma
LRF: Link retention factor
LBY: Look back years
nMOFs: Nanoscale metal–organic frameworks
NIR: Near-infrared
PB: Prussian blue
PCM: Phase-change material
PD-L1: Programmed death-ligand 1
PDT: Photodynamic therapy
POD: Peroxidase
PTT: Photothermal therapy
PVA: Polyvinyl alcohol
RNAi: RNA interference
ROS: Reactive oxygen species
O₂·⁻: Superoxide anions
TLS: Total link strength
TME: Tumor microenvironment
TNBC: Triple-negative breast cancer
USP8: Ubiquitin-specific peptidase 8
WoSCC: Web of Science Core Collection
X-PDT: X-ray-induced photodynamic therapy
