# Supplementary material for: Landscape and evolutionary trends of nanotechnology in cancer radiosensitization
Source: Discov Nano. 2026 Aug 4;21(1):375. doi: 10.1186/s11671-026-04836-8 (PMC13438096; doi:10.1186/s11671-026-04836-8)
Supplement: Supplementary file 1 [file 11671_2026_4836_MOESM1_ESM.docx]

**Systematic Analysis** **of the Landscape and Evolutionary Trends of Nanotechnology in Cancer Radiosensitization**

**Authors:**

Miao Yan^1,2,3,4*^, Yuanchao Cheng^1,2,3,4^, Quan Li^1,2,3,4^, Xvdong Zhou^1,2,3,4^, Chao Ren^1,2,3,4,5†^, Guilong Zhang ^6†^, Xicheng Song^1,2,3,4†^

**Affiliations:**

^1^Department of Otorhinolaryngology, Head and Neck Surgery, Yantai Yuhuangding Hospital, Qingdao University, Yantai, China.

^2^Shandong Provincial Key Laboratory of Neuroimmune Interaction and Regulation, Yantai Yuhuangding Hospital, Yantai, China.

^3^Shandong Provincial Clinical Research Center for Otorhinolaryngologic Diseases, Yantai Yuhuangding Hospital, Yantai, China.

^4^Yantai Key Laboratory of Otorhinolaryngologic Diseases, Yantai Yuhuangding Hospital, Qingdao University, Yantai, China.

^5^Department of Neurology, Yantai Yuhuangding Hospital, Qingdao University, Yantai, China.

^6^ School of Pharmacy, Shandong Technology Innovation Center of Molecular Targeting and Intelligent Diagnosis and Treatment, Shandong Medical and Pharmaceutical University, Yantai, China

^†^**Correspondence:**

Prof. Dr. Xicheng Song. Yantai Yuhuangding Hospital, Qingdao University, No.20, East Road, Zhifu District, Yantai 264000, China; E-mail: [drxchsong@163.com](mailto:drxchsong@163.com).

Dr. Guilong Zhang. School of Pharmacy, Shandong Technology Innovation Center of Molecular Targeting and Intelligent Diagnosis and Treatment, Shandong Medical and Pharmaceutical University, No. 346, Guanhai Road, Laishan District, Yantai 264003, China; E-mail: glzhang@bzmc.edu.cn.

Dr. Chao Ren. Yantai Yuhuangding Hospital, Qingdao University, No.20, East Road, Zhifu District, Yantai 264000, China; E-mail: [renchaotg@126.com](mailto:renchaotg@126.com).

**Figure S1.** The global annual number of citations of the publications.

**Figure S2.** Country co-authorship analysis by VOSviewer. In this overlay visualization map, each node is a country, and links between countries represent co-authorship relationships. The size of each node is proportional to the total number of publications.

 **Figure S3.** **(A)** Institution co-authorship analysis by VOSviewer. **(B)** Overlay visualization of institution co-authorship analysis by VOSviewer.

**Figure S4. (A)** Author's local impact by H-index. **(B)** The number of authors’ publications. **(C)** Authors’ collaboration network.

**Figure S5. (A)** The change trend of annual publications of the top 5 journals from 2006 to 2024. **(B)** Journals’ local impact by H-index. **(C)** Overlay visualization map of keyword co-occurrence analysis. The node color reflects the corresponding AAY according to the color gradient in the lower right. Overlay visualization map of keyword co - occurrence analysis. The node color reflects the corresponding AAY according to the color gradient in the lower right.
